# Position‐Specific Substitution in Cellulose Ethers Studied by DNP Enhanced Solid‐State NMR Spectroscopy

**DOI:** 10.1002/mrc.5535

**Published:** 2025-05-22

**Authors:** Hampus Karlsson, Arthur C. Pinon, Leif Karlson, Helena Wassenius, Frida Iselau, Staffan Schantz, Lars Evenäs

**Affiliations:** ^1^ Department of Chemistry and Chemical Engineering Chalmers University of Technology Gothenburg Sweden; ^2^ FibRe‐Centre for Lignocellulose‐Based Thermoplastics, Department of Chemistry and Chemical Engineering Chalmers University of Technology Gothenburg Sweden; ^3^ Wallenberg Wood Science Center Chalmers University of Technology Gothenburg Sweden; ^4^ Swedish NMR Centre, Department of Chemistry and Molecular Biology University of Gothenburg Gothenburg Sweden; ^5^ Nouryon Functional Chemicals AB Stenungsund Sweden; ^6^ Technical Operations, Science and Innovation, Pharmaceutical Technology & Development, Operations AstraZeneca Gothenburg Sweden; ^7^ Oral Product Development, Pharmaceutical Technology & Development, Operations AstraZeneca Gothenburg Sweden

**Keywords:** cellulose ethers, DNP, EHEC, MEHEC, solid‐state NMR, substitution

## Abstract

Ethyl hydroxyethyl cellulose (EHEC) and methyl ethyl hydroxyethyl cellulose (MEHEC) are hydrophilic cellulose ethers commonly employed as rheology modifiers in diverse industrial applications. The performance of these polymers, and their resistance to degradation by various cellulase enzymes, depends on their intricate molecular structure. Distribution of the etherifying groups, within the anhydroglucose units and along the polymer chain, is the key property to control. However, characterizing such structural properties is challenging, necessitating the development of novel analysis methods. In this study, we demonstrate the application of solid‐state nuclear magnetic resonance (NMR) spectroscopy, enhanced by dynamic nuclear polarization (DNP), for this purpose. We prove that the hydrophilic EHEC and MEHEC samples are homogenously swelled in D_2_O/H_2_O‐based radical solutions, a necessity to ensure uniform DNP enhancement throughout the material. And we illustrate how the high sensitivity enhancements obtained can be used to perform selective, *J*‐coupling‐based C1 to C2 transfer experiments to measure the fraction of substituted C2 positions in these cellulose ethers. Moreover, with further refinement, the methodology outlined in this work holds promise for elucidating C3‐specific substitution patterns.

## Graphical abstract

C1 to C2 coherence transfer for quantification of C2 substitution.

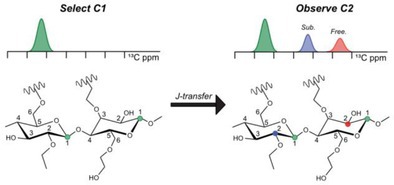

## 1 Introduction

Etherified cellulose materials can be found in many different industrial applications. By substituting the hydroxyl groups on the cellulose backbone into ether groups, functionally diverse materials can be created. Methyl cellulose (MC) and ethyl cellulose (EC) typically have applications in food and drug industry [1, 2]. Other derivatives with multiple different side chains such as methyl ethyl hydroxyethyl cellulose (MEHEC) and ethyl hydroxyethyl cellulose (EHEC) have for instance found use as rheology modifiers in areas such as the paint and construction industry. The rheology modifying effect can be reduced if the cellulose ether is attacked by cellulase enzymes and the β‐1,4‐linkage between the anhydroglucose units (AGUs) is cleaved. This can happen if a paint is contaminated with cellulase‐producing microorganisms, which may come from equipment and surroundings [3]. For such enzymatic attacks, the degree of substitution (DS) and the distribution of the etherifying side chains along the cellulose backbone will affect the accessibility for cleavage and thus also the biostability [4, 5]. DS refers to how many of the hydroxy groups on the AGU in the cellulose backbone that have reacted (on average along the polymer); it takes on a numerical value between 0 and 3, where a DS = 3, indicates that all three hydroxy groups have been substituted [6]. The DS value is used for substituents, such as methyl and ethyl groups, that blocks the hydroxy groups from participating in further nucleophilic attacks. When the hydroxy groups on cellulose instead are reacted with a molecule (such as ethylene oxide [EO] for instance), that in turn generates a new nucleophilic moiety that can react again, the sidechains can in theory become infinitely long. For such substituents, one reports the molar substitution (MS), which is the number of moles of the substituent per AGU [7]. Because of the intricate relationship between DS/MS and properties of the final modified cellulose polymer, control of the substitution reactions and the possibility to measure DS and MS and side chain distribution becomes of outmost importance. Traditionally, these types of properties have been investigated with a variety of chemical, chromatographic and spectroscopic methods such as gas chromatography (GC), high‐performance liquid chromatography (HPLC), size exclusion chromatography (SEC), mass spectrometry (MS) and nuclear magnetic resonance (NMR) spectroscopy [6, 8, 9]. However, these existing methods are all associated with drawbacks of their own. GC methods for analyzing hydroxyethyl‐based side chains, might for instance include tedious acid‐based degradation steps [10], liquid‐state NMR is limited in what concentrations that are feasible to use, and ordinary ^13^C‐detected room temperature solid‐state NMR is slow due to the low abundance of ^13^C. Dynamic nuclear polarization (DNP), on the other hand, is an NMR technique used to enhance NMR signals and thus speed up NMR experiments. The DNP method is most often used within the solid‐state NMR context, typically in combination with magic angle spinning (MAS) and forms an attractive option for studies of cellulose ethers.

DNP was initially explored in the 1950s and it has re‐emerged as method in the recent decades as commercial DNP equipment have become more widely available [11]. During a DNP‐enhanced solid‐state NMR experiment, the NMR sample is irradiated with microwaves in the presence of unpaired electrons (i.e., radicals) at low temperatures (typically 100 K). This leads to a transfer of the high spin polarization of the unpaired electrons to the atomic nuclei of the sample and enhances their NMR signal by orders of magnitude [12, 13, 14, 15]. Before the DNP‐NMR experiment, radicals are added to the sample with a solution containing organic molecules with unpaired electrons; it is moreover required that the solution wets the polymer material. AMUPol [16] and TEKPol [17] are examples of well‐established radicals for DNP usage. Cryoprotecting agents such as glycerol‐d8 [18] or DMSO‐d6 [19] might also be added to the solution; although radical solutions without cryoprotecting agents have been shown to give high DNP sensitivity enhancement in cellulose materials [20, 21, 22]. We do not add any cryoprotecting agent to the radical solutions in our study, as the sample material, EHEC and MEHEC readily swell in contact with water‐based solutions. For cellulosic materials, it is also an option to evaporate the radical solvent in a desiccator to get the radical adsorbed on the surface of the solid [23].

**FIGURE 1:**
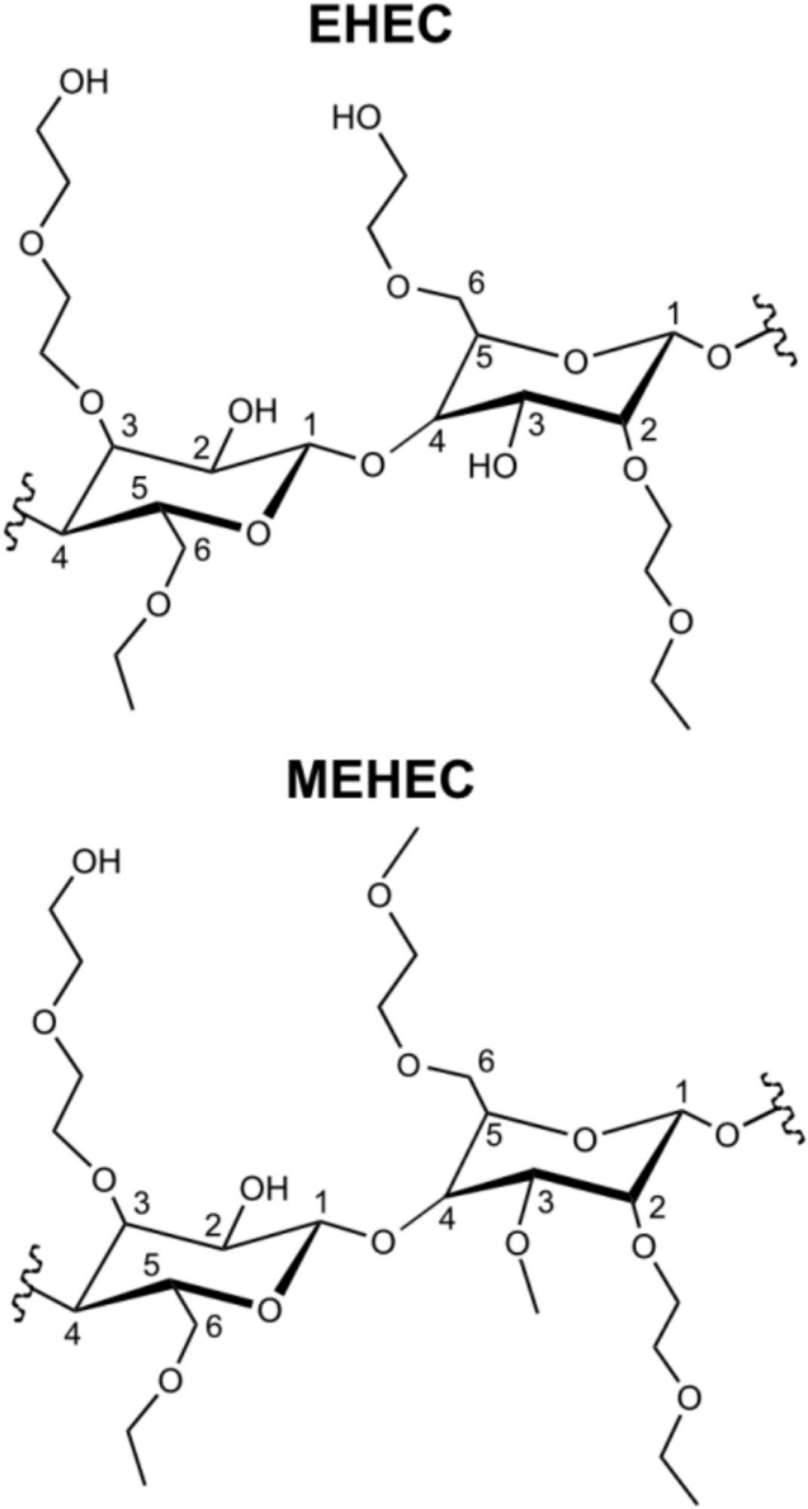
Molecular structures of possible repeating units of ethyl hydroxy ethyl cellulose (EHEC) and methyl ethyl hydroxy ethyl cellulose (MEHEC). In MEHEC, additional methyl groups are introduced, making molecular structure more complex.

DNP‐enhanced solid‐state NMR has been successfully deployed before to study cellulose ethers and cellulose‐related materials [20, 23, 24, 25, 26, 27, 28, 29]. Especially, studies such as [20], focusing on the molecular regioselectivity of substitution in cellulose ethers, are important to contextualize our study. The study by [20] focuses on MC while our study focuses on EHEC and MEHEC, which are hydrophilic cellulose ethers with significantly more heterogeneous side chains (Figure 1) than MC, and in addition, with an industrial large‐scale synthesis origin. Target in our study is the substitution at the C2 position of the AGUs, and we hypothesize that it is possible to use DNP enhanced solid‐state NMR and selective, 1D, *J*‐coupling‐based correlation experiments for this site‐specific quantification.

## 2 Experimental

### 2.1 Sample Material and Radical Solution

EHEC and MEHEC samples were provided by Nouryon (Stenungsund, Sweden). All samples were of industrial, large‐scale synthesis origin and with natural abundance levels of the ^1^H and ^13^C isotopes. Samples were classified as either biostable or non‐biostable based on their production method and further verified by the reduction of viscosity of their water solutions in the presence of cellulase. These cellulase resistance assays were done by measuring the viscosity after 60 min of cellulase exposure. A 1 wt % polymer solution in 50‐mL buffer (pH 5.5) was stirred at 425 rpm in a Rheomat 108 viscometer and kept at a temperature of 35°C in presence of 0.375 units of cellulase EC3.2.1.4 from *Aspergillus niger* (Sigma‐Aldrich). The solutions were stirred for 60 min after which the viscosity was noted as a fraction of the starting viscosity. The obtained values were considered a quantitative measure of the biostability of the polymer, and from here on, these values are referred to as %S60 values in the text. MS of EO and DS of ethyl groups were determined by GC following the degradation of the products with HBr in glacial acetic acid [10]. Determination of DS for methyl groups was done with a slightly modified method, and the degradation was carried out with HI in glacial acetic acid instead of HBr. The given values, from here on referred to as MS_EO_, DS_me_, and DS_et_ correspond to the average numbers of EO, methyl, and ethyl groups per sugar unit, respectively. Radical solutions for DNP NMR experiments consisted of 12‐mM AMUPol (cortecnet.com) dissolved in D_2_O:H_2_O (9:1 by volume) using D_2_O (99.8% D, ARMAR Isotopes) and MilliQ water (resistivity 18.2 MΩ·cm at 25°C. ^13^C labeled sodium formate (Sigma‐Aldrich) was added during relaxation experiments to measure *T*_DNP_ (*longitudinal buildup time of proton z‐magnetization under DNP conditions*) of the radical solution. Typically, some 20 mg of cellulose ether material was wetted or swelled in 40 μL of the radical solution prior to packing into a 3.2‐mm DNP NMR sapphire rotor with a PTFE plug and a VESPEL cap.

### 2.2 DNP System

DNP NMR experiments were performed on a 400‐MHz Bruker Ascend DNP magnet. The system was equipped with a 263‐GHz gyrotron and a 3.2‐mm LTMAS DNP probe. Experiments were performed with MAS rates ranging from 8 to 10 kHz; for all *J*‐coupling‐based experiments, MAS rates of 10 kHz were used. The temperature measured on the output gas from the LTMAS probe was ~104 K for experiments run with the microwaves on.

### 2.3 NMR Experiments

2D refocused INADEQUATE experiments [30, 31] were acquired with 64 points in the indirect dimension and 1k points in the direct dimension and 512 scans. Four‐millisecond long delays for *J*‐evolution in the spin‐echoes were used and the relaxation delay between scans was set to 1.3 *× T*_DNP_ ≈ 4.3 s. 1D selective, *J*‐coupling‐based experiments were carried out in the following manner: After a nonselective cross‐polarization (CP) [32] transfer, a selective flip‐back pulse (1‐ms Gaussian pulse with 10% truncation level) was applied on the C1 carbon to flip the magnetization back to +z. After the flip‐back, a 2‐ms long continuous wave (CW) irradiation on the ^1^H channel followed to relax away remaining transverse ^13^C coherences (z‐filter). The ^1^H CW was matched with the MAS rate (rotational resonance) [33]. Thereafter, the same selective, truncated Gaussian pulse was applied on C1 to flip the magnetization down again, whereby a perfect‐echo [34, 35, 36] for coherence transfer via *J*‐coupling followed (see for instance Figure 4, further down). For the selective flip‐down pulse after the z‐filter, the phase was x,x,x,x,x̄, x̄, x̄, x**¯**; for the perfect‐echo, the pulse phases were x, −y, and x for the π, π/2, and π pulse. The phase of the receiver was ȳ, ȳ, ȳ, ȳ, y,y,y,y. Control experiments with the π/2 pulse in the perfect‐echo removed were performed to quantify artefact signals from the shaped pulses. After Fourier transform, phasing, and baseline correction, the spectra from these control experiments were subtracted from the actual transfer experiments and the resulting difference spectra were integrated over the C2 peak. Also, these experiments were performed with the relaxation delay between scans set to 1.3 *× T*_DNP_ and typically between 2 and 4k scans. Longitudinal ^1^H relaxation times were measured with standard ^1^H detected saturation recovery pulse sequence and with a pulse sequence with a CP transfer step at the end for ^13^C detection. All NMR data were processed with Topspin 4.1.1 and Python 3 scripts; the “Nmrglue” [37] python module was used during the processing of data from the 1D transfer experiment, in order to plot in detail and study the impact of baseline corrections and phasing. Relaxation data from saturation recovery experiments was fitted to the stretched exponential function [38].

### 2.4 Electron Microscopy

Scanning electron microscopy (SEM) images of the cellulose ether samples were acquired on a Zeiss Ultra 55 FEG scanning electron microscope. Acceleration voltage was set to 3.0 kV and the working distance was 11 mm during imaging. The samples were coated with a 10‐nm gold layer with a Leica ACE600 sputter coater machine.

## 3 Results

### 3.1 DNP Signal Enhancement in Hydrophilic Cellulose Ethers

**FIGURE 2:**
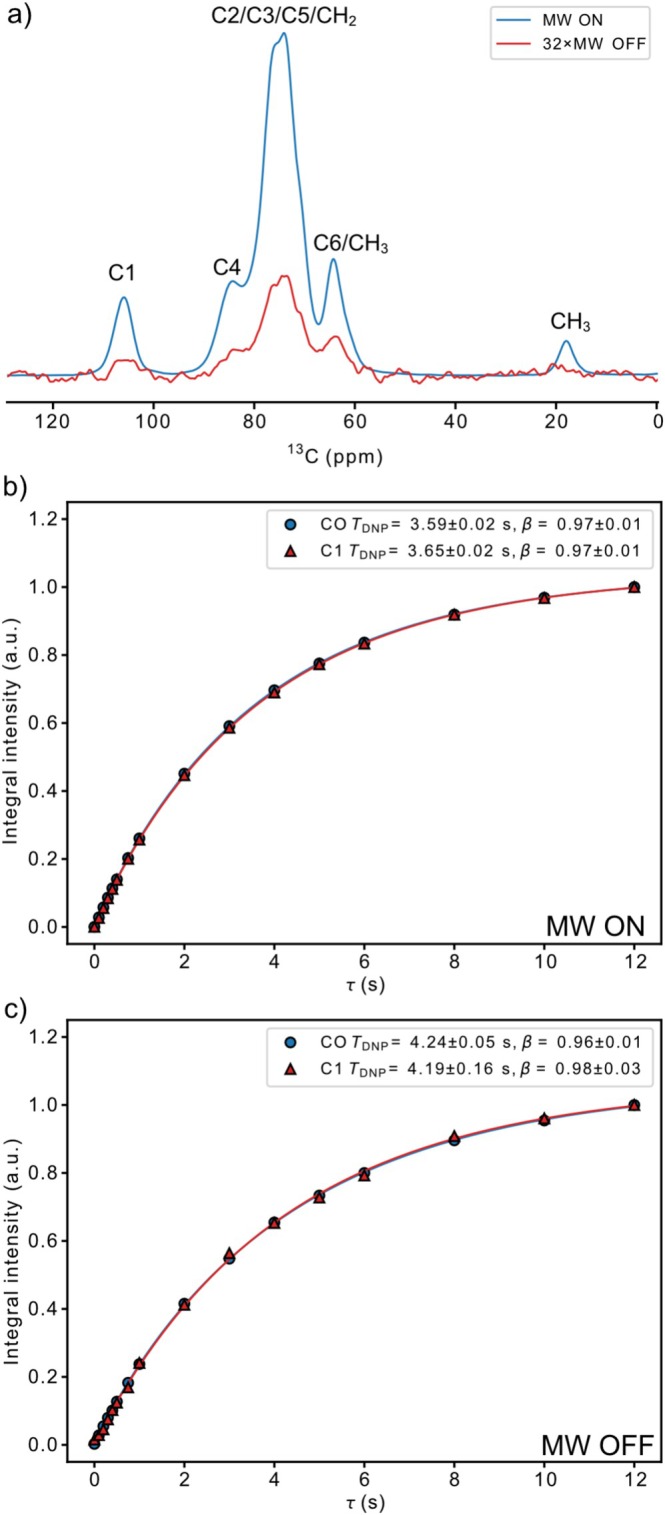
DNP enhanced ^13^C CP/MAS and ^13^C detected ^1^H saturation recovery data for the EHEC2 sample swelled in 12‐mM AMUPol D_2_O:H_2_O (9:1) radical solution. (a) ^13^C CP/MAS spectra illustrating the large sensitivity enhancement when the microwaves are turned on/off. (b) ^13^C detected ^1^H saturation recovery data acquired with microwaves turned on, sodium formate was added to the radical solution, and the curves illustrate the practically identical buildup time detected on the carbonyl signal (CO) of the formate ion and the C1 carbon (C1) of EHEC. (c) ^13^C detected ^1^H saturation recovery data acquired with microwaves turned off, the buildup times differ from the ones observed with the microwaves turned on but are also here, practically the same for CO and C1.

The hydrophilic cellulose ethers EHEC and MEHEC showed high DNP sensitivity enhancements (*ε*_DNP_) in D_2_O/H_2_O‐based radical solutions. This is exemplified by the ^13^C CP/MAS spectra of sample “EHEC2” (Figure 2a), which is a biostable cellulose ether and that showed a *ε*_DNP_ ≈ 120, here reported as the ratio of the spectrum integrals acquired with microwaves on/off. We choose to report the sensitivity enhancement like this here, but it should be kept in mind that the signal in the microwave off spectrum, likely is significantly reduced (up to 60%) due to depolarization phenomena associated with the AMUPol usage [39]. Thus, our polarization gain in terms of Boltzmann distributions would be around a factor of 50 for our DNP experiments performed with the microwaves turned on.

This strong signal enhancement can be attributed to the fact that the EHEC and MEHEC samples readily swell in the D_2_O:H_2_O‐based radical solutions used in the experiments. The size of the industrially produced cellulose ether particles, when they are intact, is usually several tens of μm and aggregates thereof much larger (see Figure S1 for exemplifying SEM pictures of EHEC2). Such large particle size would lead to negligible DNP signal enhancements if the particles remained completely intact in the radical solution, as only the surface layer of the particles would become hyperpolarized. This size range ~20–100 μm practically makes spin diffusion phenomena [40, 41] irrelevant for explaining the enhancements observed. ^1^H spin diffusion constants for our samples would be expected to be around 10^−15^ m^2^/s [42], this fact combined with that the ^1^H *T*_1_ relaxation time of the dry EHEC2 powder at 100 K is 13.3 + 1.7 s (see Figure S2) and that most of our relaxation delays are less than 10 s means that ^1^H polarization never spin diffuses longer than ~0.1 μm. Thus, swelling in the radical solution is likely the dominant factor determining enhancement. The question is though if swelling is homogeneous in the EHEC/MEHEC samples and if all parts are in good contact with the radical solution. This can be assessed by analyzing the saturation recovery data (Figure 1). The relaxation data acquired with the microwaves turned on (Figure 1b) show that the carbonyl signal of the sodium formate (i.e., known to be completely dissolved in the D_2_O/H_2_O‐based radical solution) has the same *T*_DNP_ as the C1 signal of EHEC. This would be the case if the EHEC was homogenously swelled, but it would also be the case if only a small fraction of the outermost layer of an EHEC‐particle would be swelled. In such case, the enhanced signal from the swollen fraction of EHEC would dominate relaxation curve, and one would observe a *T*_DNP_ detected on the EHEC, very close to that observed on the formate in the radical solution, thus the fact that *T*_DNP_ is equal for CO and C1 in Figure 1b does not prove that EHEC is homogenously swollen. However, in the relaxation data acquired with microwaves turned off (Figure 1c), the situation is different, then the swelled part is expected to relax fast due to paramagnetic relaxation enhancement effects from the radical [43, 44, 45], but it will not be enhanced (instead, slightly reduced due to depolarization). Thus, for the relaxation data acquired with the microwaves turned off (Figure 1c), *T*_DNP_ observed on C1 of EHEC will not approach that of CO in the radical solution unless the EHEC is fully, homogenously swelled in the radical solution. We prove this by modelling the relaxation behavior of the EHEC system according to a biexponential model with different amounts of swelling (Supplementary discussion and Figure S3).

### 3.2 Investigating C2 Substitution

**FIGURE 3:**
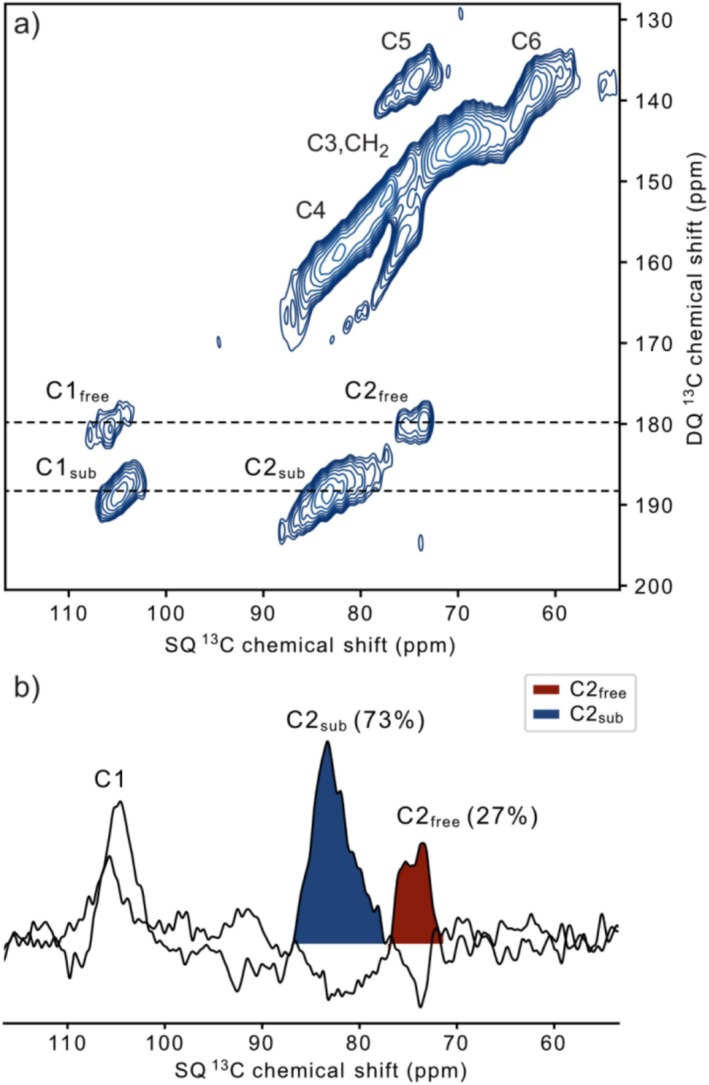
^13^C–^13^C DNP‐enhanced refocused INADEQUATE spectrum of EHEC2. (a) 2D spectrum, it is easy to spot the C1/C2 correlations and chemical shift change of the substituted and free C2 positions, located around 75 and 84 ppm in the direct (horizontal) single quantum (SQ) dimension and around 180 and 189 ppm in the indirect (vertical) double quantum (DQ) dimension. (b) 1D slices of the rows in the 2D spectrum that correspond to the C1/C2 correlations, where peak integrals are indicative of substitution at the C2 position.

The strong DNP sensitivity enhancements that we observed in these materials opened up the possibility of doing correlation experiments in a time‐efficient manner. We were particularly interested in looking into the site‐specific substitution on the AGU. It is well‐known that both the DS and type of substituent affect the biostability of cellulose derivatives [5] and some studies [46] have indicated that, especially C2 and C3 substitution are more efficient in protecting against cellulose degradation. Because of this, methods that can inform on C2 substitution are valuable. The NMR chemical shift of the C2, C3, and C6 carbon of the AGU in cellulose is typically shifted 1–10 ppm downfield upon substitution [47]. This chemical shift change can be taken advantage of in order to estimate substitution. However, in nonsubstituted cellulose both C2, C3, and C5 end up overlapping in the mid 70‐ppm region [48]. In the heavily substituted EHEC and MEHEC systems, the chemical shift of C2 and C3 starts to vary due to substitution. Additional overlap is also introduced by the signals from the carbons in the CH_2_ groups in the substituting side chains (ethyl and hydroxyethyl), and the highly amorphous character of these cellulose ethers due to the extensive substitution, further contributes to chemical shift variations. Thus, there is a considerable overlap in the 70–90‐ppm region in the CP/MAS spectra (such as Figure 1a) of these polymer systems. One way to get around the chemical shift overlapping problem of the 1D spectra is to use a 2D correlation experiment. The *J*‐coupling‐based, refocused INADEQUATE experiment [30, 31] is common, and several examples of its application to cellulosic materials under DNP‐conditions exist [20, 22, 25, 29, 49]. Figure 3a shows a DNP enhanced, ^13^C–^3^C refocused INADEQUATE spectrum of EHEC2. It is clear in this 2D spectrum that there are two different correlations originating from the substituted and free C2 positions. Figure 3b exemplifies how for instance the 1D slices corresponding to these correlations can be analyzed to yield an estimate of the C2 specific substitution. Several factors will affect the accuracy of such quantifications but that will be addressed in the next Section 3.3.

**FIGURE 4:**
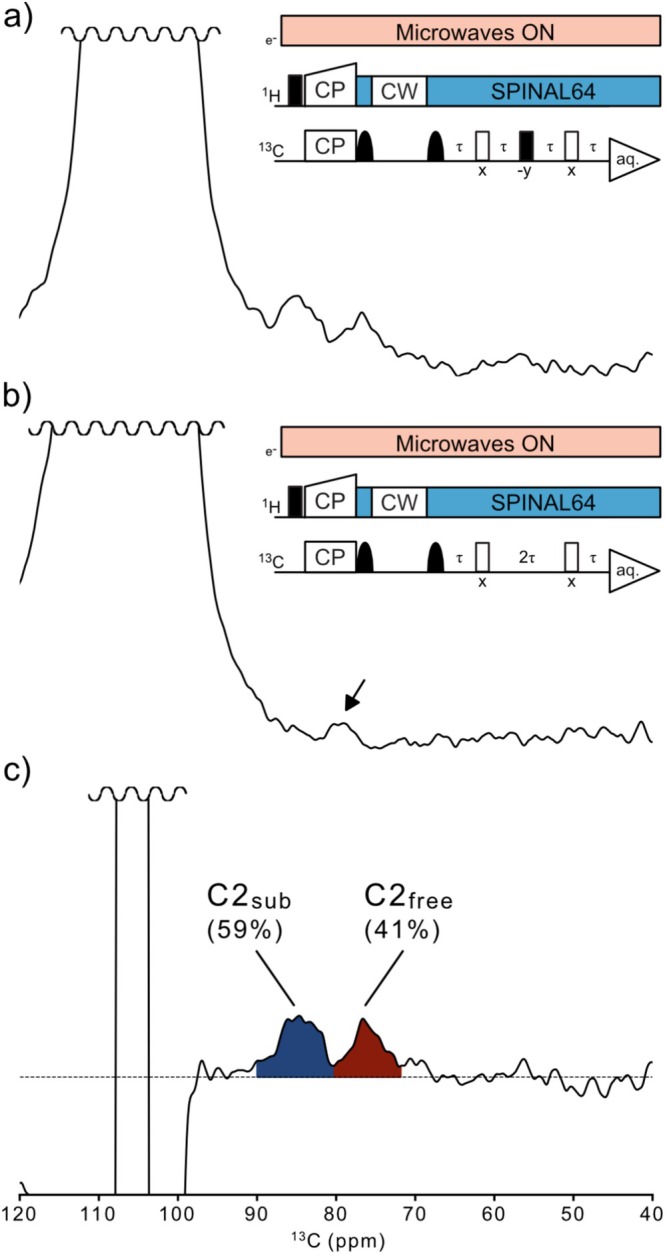
1D, *J*‐coupling‐based correlation experiment for quantification of C2 substitution. (a) Spectrum from selective transfer experiment of the EHEC2 sample at MAS = 10 kHz, T = 104 K, and 12‐mM AMUPol D_2_O:H_2_O (9:1) radical solution and 2048 scans/2 h experimental time. The pulse sequence with selection step and perfect echo is shown. (b) Spectrum from the control experiment in which the π/2 transfer pulse has been removed from the perfect echo. The spectrum thus only contains *T′*_2_ decayed artifact signal indicated by the small black arrow. (c) Difference spectrum (transfer experiment (a)—control experiment (b)), here, the signals are assumed to come only from the *J*‐coupling transfer and are thus integrated. The signal in spectral region 90.1–80.4 ppm is considered to originate from substituted C2 and the signal in the 80.4–71.9‐ppm region from free, nonsubstituted C2. The percentage of the integral relative to the integral of the whole region is shown. A dashed line indicates the baseline.

Performing the DNP‐enhanced, ^13^C–^13^C refocused INADEQUATE experiment on cellulose samples with natural abundance levels of ^13^C can be lengthy and take several days. The spectrum shown in Figure 3a was acquired in around 24 h, thanks to the high sensitivity enhancement (*ε*_DNP_ ≈ 120). But since acquisition time for this type of experiment is lengthy and C2 substitution was our primary interest, we instead devised the selective 1D experiment illustrated in Figure 4. Using a selective 1D experiment saves time compared to a 2D experiment. The experiment outlined in Figure 4 (see details in Section 2) uses selective pulses and a z‐filter for selecting C1, followed by transfer from C1 to C2. Initially, it was considered to use a selective CP transfer step to select C1, but it was soon realized that this is only feasible to do under fast MAS conditions [50]. Because of this, the strategy with a nonselective CP transfer, followed by the z‐filter was chosen, a solution based on other examples from the solid‐state NMR literature [42, 51]. However, the chosen z‐filter approach is associated with both considerable signal losses during the shaped pulses and some artifact excitation (see Figure S4 for the properties of the selective pulses and the z‐filter). The artefact excitation necessitated the control experiment in Figure 4b and the procedure of subtracting the control spectra (difference spectroscopy). After selection of the C1 signal, a perfect‐echo was used for *J*‐coupling mediated coherence transfer from C1 to C2. This choice was based on familiar examples from the solution state NMR literature [52, 53]. Usage of the perfect‐echo means that the large signal from all non‐coupled C1 nuclei will give signal in the spectrum as there is no means of coherence order filtering with the perfect‐echo. The alternative would have been to use a pulse sequence fully analogous to the 2D refocused INADEQUATE experiment, i.e., with two π/2 pulses between the spin‐echoes (and no time evolution in the indirect dimension), create a double quantum coherence between them and then use a phase cycling scheme to select it. This was tested to some extent, but performance was poor on our cellulose samples under DNP‐conditions (Figure S5). Another drawback of this approach would have been that the double quantum selective phase cycle only leads to transfer for half of the scans, which matters in the case of doing a 1D selective experiment, only starting with magnetization on C1. Because of the poor performance on our cellulose samples under DNP‐conditions and the issue with transfer for only half of the scans, the simpler perfect‐echo was chosen.

The experiment shown in Figure 4 was thus tested on a series of EHEC and MEHEC samples and the results of these experiments are summarized in Table 1, where also biostability data and DS/MS values of the substituents are shown. Ethers that were known to be biostable or non‐biostable were included in the test sample set to see if the NMR method could discern any clear difference in C2 substitution between these two categories of ethers.

**TABLE 1:** Biostability, %S60, C2 site‐specific substitution, and DS/MS values for the series of EHEC and MEHEC samples investigated. The second column shows if the ether is classified as biostable or not, based on production method. The third column shows %S60 (remaining viscosity after 60‐min cellulase exposure). The fourth column shows measured C2 substitution from the 1D *J*‐correlation experiments, errors represent one standard deviation and were estimated from sample EHEC1, the only sample with triplicate measurements. The three rightmost columns show the DS/MS for methyl, ethyl, and ethylene oxide groups obtained from gas chromatographic methods. Notice that also the EHEC samples contain some substituted CH_3_ groups, this is due to production technical factors.

Name	Classification	%S60 pH 5.5, 35°C	% C2 substituted (90.1–80.4 ppm)	DS_me_	DS_et_	MS_EO_
EHEC1	Non‐biostable	63.7	53 ± 12	0.26	0.64	1.85
EHEC2	Biostable	75.8	59 ± 13	0.16	0.70	2.07
EHEC3	Biostable	81.4	54 ± 12	0.00	0.74	2.20
MEHEC1	Non‐biostable	55.3	53 ± 12	0.30	0.42	1.52
MEHEC2	Biostable	81.7	63 ± 14	0.42	0.37	1.82

The NMR spectra from the 1D correlation experiments underlying the results in Table 1 can be found in Figure S6. As can be seen in Table 1, the measured C2 substitution values do vary between the samples, but there is no clear difference in the C2 substitution between the biostable and non‐biostable ethers. Between the two MEHEC samples, there is an indication of a difference, but this is a too limited number of samples to draw any certain conclusions from. Yet, the method shown in Figure 4 offers a less time‐consuming way than the 2D INADEQUATE experiment for obtaining semi‐quantitative values of the C2 substitution. The three rightmost columns of Table 1 provide the DS/MS values for the substituents determined by chemical, non‐NMR methods. These data are included as complementary information and to highlight the complexity of these polymers and illustrate that despite differences in substitution properties, the polymers can have similar biostability performance. Our method provides partial information about these polymers, it can tell about C2 substitution in a reasonably time efficient and accurate manner, but it cannot inform on what substituent there is on C2. To fully elucidate this and all the factors affecting substitution and biostability, it is likely that data from complementary methods are necessary. Bos et al. [54] presented an LC–MS‐based method developed on a related set of cellulose ethers. Such methods can provide information on the distribution of side chain lengths and may complement data from NMR methods such as the one outlined in Figure 4.

### 3.3 Accuracy of the C2 Substitution Values

**FIGURE 5:**
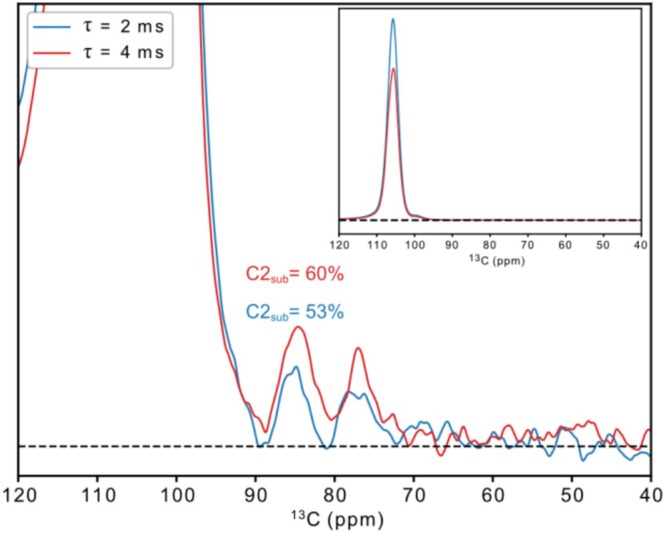
DNP‐enhanced spectra from the 1D transfer experiment (no control spectra subtracted) acquired on the MEHEC2 sample with different τ delay in the spin‐echoes, showing a clear buildup of signal for the 4‐ms delay. The buildup of signal and small difference in proportion of the C2_sub_ correlation signal supports the claim that differences in *J*_C1C2_ and *T′*_2_ are not a major problem affecting the results. The inner panel shows a zoomed‐out view of the spectra, illustrating the *T′*_2_ decay of the large C1 signal.

In the previous section, we suggest the usage of the selective 1D *J*‐coupling‐based experiment (Figure 4) to obtain correlation signals of the C2 carbons of the AGU, signals that can be integrated to get a measure of the fraction of substituted C2 positions. Despite the beneficial sensitivity enhancement properties of these hydrophilic cellulose ethers in D_2_O/H_2_O based radical solutions, the signal‐to‐noise ratios associated with these types of experiments are in general very low. These low signal‐to‐noise ratios make the obtained values for C2 substitution semiuantitative in nature. Even so, the data we present here (Table 1, Figures S6 and S7) are acquired on industrially produced cellulose ethers with complex side‐chains and no degradation steps or similar are included in the sample preparation step; thus, the DNP‐enhanced solid‐state NMR method allows the study of the samples intact and with technically no limit in degree of polymerization. In addition to a low signal‐to‐noise ratio, we have performed these experiments as difference spectroscopy, subtracting the spectra from the control experiments from the actual transfer experiments. This was done because of the aforementioned artefact signals that originate from the shaped pulses used in the z‐filter (Figure S4). This process, the phasing, baseline correction, and subtraction of the spectra, is a very sensitive procedure to perform at these low signal‐to‐noise ratios and unavoidably opens up for some subjectivity. To obtain the numbers presented in Table 1, typically, the regions between the large C1 signals and their first sidebands were used to perform a fifth‐order polynomial baseline correction of the transfer and control spectra (Figure S8), after this baseline correction, the spectra from the control experiments were subtracted. Potential differences in *J*_C1C2_ and *T′*_2_ between substituted and free C2 may also impact the magnitude of the correlation peak integrals and the resulting C2 substitution values. However, Figure 5 illustrates the buildup of peak intensity in the 1D transfer experiment for two different τ delays for the MEHEC2 sample. We interpret the small difference in peak integral proportions as an indication that differences in *J*_C1C2_ and *T′*_2_ are not a major problem affecting the results. Thus, we claim that if the 1D correlation experiments are done carefully and with good control of potential artefact signals, processing and peak integration, reliable semi‐quantitative values of C2 substitution can be obtained.

## 4 Conclusion

In this work, we have shown how a selective 1D, *J*‐coupling‐based correlation experiment can be used to quantify the C2 substitution in hydrophilic cellulose ethers under DNP MAS conditions. The results from the selected set of cellulose ethers investigated exemplify how such an experiment could contribute to obtain information about substitution and biostability of cellulose ethers. We also believe that with some further development, and in combination with higher availability of DNP equipment with faster MAS rates combined with DNP enhancement, it could most possibly be attainable to deconvolute the C2 correlation signals and make indirect conclusions about substitution on C3. Under such conditions the experiment could be used to quantify substitution on both C2 and C3 in intact, industrially relevant cellulose ethers in one single experiment.

## Supporting information


**Figure S1.** Scanning electron microscopy images of EHEC2. Intact particles are several tens of μm, and aggregates are larger than 100 μm. Red area in (a) identical to (b).
**Figure S2.** ^1^H detected ^1^H saturation recovery data of dry EHEC2 powder at T = 100 K, MAS = 8 kHz.
**Figure S3.** Fitting of synthetic relaxation datasets, generated with the biexponential model for swelling of the EHEC. The curves in the middle column illustrate that already at 2.5 μm/18% vol. of swelling, the relaxation becomes dominated by the enhanced, swelled part, and the fitted *T*_B_ (*T*_DNP_) value approaches that of the radical solution. The rightmost column shows that when the microwaves are turned off, complete swelling of the EHEC is necessary for the buildup time observed on the EHEC to equal that of the radical solution. This proves that for the relaxation curves of CO and C1 to be equal in Figure 2c in the main text, EHEC needs to be fully swelled.
**Figure S4.** Properties of the z‐filter and selective pulses. (a) Comparison of the DNP‐enhanced ^13^C CP/MAS spectrum and the CP + z‐filter part (pulse sequence shown) of the transfer experiment. The dashed spectrum shows C1 selection; the intensity of the C1 signal is ~50% of that in the CP/MAS spectrum. This is most likely due to relaxation during the two selective pulses in the z‐filter. (b) Artefact excitation from different soft pulses tested in the z‐filter. (c) Control experiment in which the selective pulses in the z‐filter have been positioned +30 ppm relative to the C1 signal. As can be seen, some C1 artefact signal is generated. The purpose of this control experiment is to exclude that dipolar‐mediated transfer phenomena plays a role in the artefact signals, this since the z‐filter basically is a DARR^3^ type mixing element.
**Figure S5.** Tests of selective 1D sequences on isoleucine and EHEC. (a) Room temperature transfer experiment tests of ^13^C/15N enriched isoleucine. Here, it is easy to select the well‐resolved CO signal at 176 ppm and transfer to C5 with perfect echo (red) double‐quantum selective phase cycle (blue) experiment. (b) Product operators at different positions in the pulse program, notice only transfer to S‐spin/spin 2 for half of the scans, in good agreement with peak intensity in (a) panel. (c) Test of blue pulse sequence on EHEC2, here, under DNP‐conditions, the phase cycle fails to suppress artefacts and generates a lot of artefact signal; hence the simpler perfect‐echo was chosen for transfer.
**Figure S6.** DNP‐enhanced ^13^C correlation spectra from the 1D, selective transfer experiments used for C2 quantification, tested on the different EHEC samples. Column 1 shows the spectra from the actual transfer experiment with the baseline correction used. Second column shows the spectra from control experiments, where hints of artefact excitation at 80 ppm can be seen; baseline correction is shown also here. The third column shows the subtraction/difference spectrum. The fourth column shows the integrated areas for C2 quantification and corresponding C2 substitution degrees in percent, the values that underlie Table 1 in the main article.
**Figure S7.** Spectra from the 1D correlation experiment, same as Figure S6 but here for the MEHEC samples.
**Figure S8.** Example of baseline correction of the spectrum from the 1D transfer experiment of the EHEC2 sample. (a) Close up view, fifth order polynomial (dashed line) is fitted to the red marked regions between the large C1 signal and the first spinning sidebands and is used as baseline correction. (b) Full view of the spectrum.

## Data Availability

NMR data is available on zenodo: https://doi.org/10.5281/zenodo.14269731.
