# Supplementary material for: Position‐Specific Substitution in Cellulose Ethers Studied by DNP Enhanced Solid‐State NMR Spectroscopy
Source: Magn Reson Chem. 2025 May 22;63(8):560–8. doi: 10.1002/mrc.5535 (PMC12223920; doi:10.1002/mrc.5535)
Supplement: Supplementary file 1 — Figure S1. Scanning electron microscopy images of EHEC2. Intact particles are several tens of μm, and aggregates are larger than 100 μm. Red area in (a) identical to (b). Figure S2. 1H detected 1H saturation recovery data of dry EHEC2 powder at T = 100 K, MAS = 8 kHz. Figure S3. Fitting of synthetic relaxation datasets, generated with the biexponential model for swelling of the EHEC. The curves in the middle column illustrate that already at 2.5 μm/18% vol. of swelling, the relaxation becomes dominated by the enhanced, swelled part, and the fitted T B (T DNP) value approaches that of the radical solution. The rightmost column shows that when the microwaves are turned off, complete swelling of the EHEC is necessary for the buildup time observed on the EHEC to equal that of the radical solution. This proves that for the relaxation curves of CO and C1 to be equal in Figure 2c in the main text, EHEC needs to be fully swelled. Figure S4. Properties of the z‐filter and selective pulses. (a) Comparison of the DNP‐enhanced 13C CP/MAS spectrum and the CP + z‐filter part (pulse sequence shown) of the transfer experiment. The dashed spectrum shows C1 selection; the intensity of the C1 signal is ~50% of that in the CP/MAS spectrum. This is most likely due to relaxation during the two selective pulses in the z‐filter. (b) Artefact excitation from different soft pulses tested in the z‐filter. (c) Control experiment in which the selective pulses in the z‐filter have been positioned +30 ppm relative to the C1 signal. As can be seen, some C1 artefact signal is generated. The purpose of this control experiment is to exclude that dipolar‐mediated transfer phenomena plays a role in the artefact signals, this since the z‐filter basically is a DARR3 type mixing element. Figure S5. Tests of selective 1D sequences on isoleucine and EHEC. (a) Room temperature transfer experiment tests of 13C/15N enriched isoleucine. Here, it is easy to select the well‐resolved CO signal at 176 ppm and trans [file MRC-63-560-s001.pdf]

Supporting Information

Position-specific substitution in cellulose ethers studied by DNP enhanced solid-state NMR spectroscopy

Hampus Karlsson[1,2,3], Arthur C. Pinon[4], Leif Karlson[2,5], Helena Wassenius[2,5], Frida Iselau[2,6,7], Staffan Schantz[2,4,7], and Lars Evenäs[1,2,3*]

[1] Department of Chemistry and Chemical Engineering, Chalmers University of Technology, Gothenburg, Sweden

[2] FibRe-Centre for Lignocellulose-based Thermoplastics, Department of Chemistry and Chemical Engineering, Chalmers University of Technology, Gothenburg, Sweden

[3] Wallenberg Wood Science Center, Chalmers University of Technology, Gothenburg, Sweden

[4] Swedish NMR Centre, Department of Chemistry and Molecular Biology, University of Gothenburg, Sweden

[5] Nouryon Functional Chemicals AB, Stenungsund, Sweden

[6] Technical Operations, Science and Innovation, Pharmaceutical Technology & Development, Operations, AstraZeneca, Gothenburg, Sweden

[7] Oral Product Development, Pharmaceutical Technology & Development, Operations, AstraZeneca, Gothenburg, Sweden

*Email: lars.evenas@chalmers.se

Table of Contents

SEM pictures, EHEC 3

T_1 relaxation time of dry EHEC powder 4

Supplementary discussion, swelling in D_2O/H_2O radical solutions 5

Simulating swelling and relaxation in EHEC 6

Properties of the z-filter and selective pulses 8

Tests of 1D selective sequences on 13C/15N isoleucine 9

C2 quantification spectra 10

Further example, baseline correction of 1D correlation spectrum 13

References 14

SEM pictures, EHEC

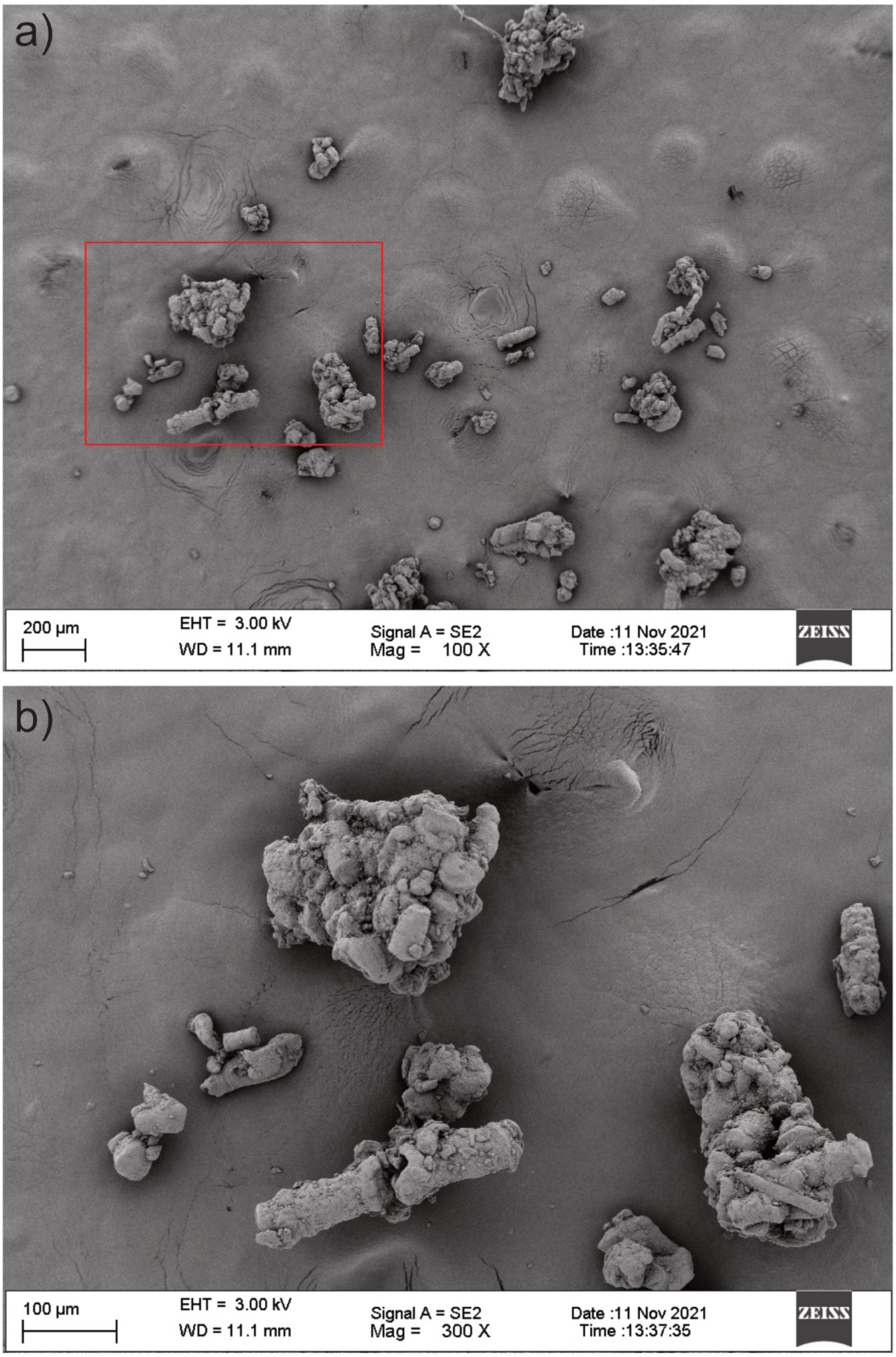

FIGURE S1. Scanning electron microscopy images of EHEC2. Intact particles are several tens of µm and aggregates are larger than 100 µm. Red area in (a) identical to (b).

T_1 relaxation time of dry EHEC powder

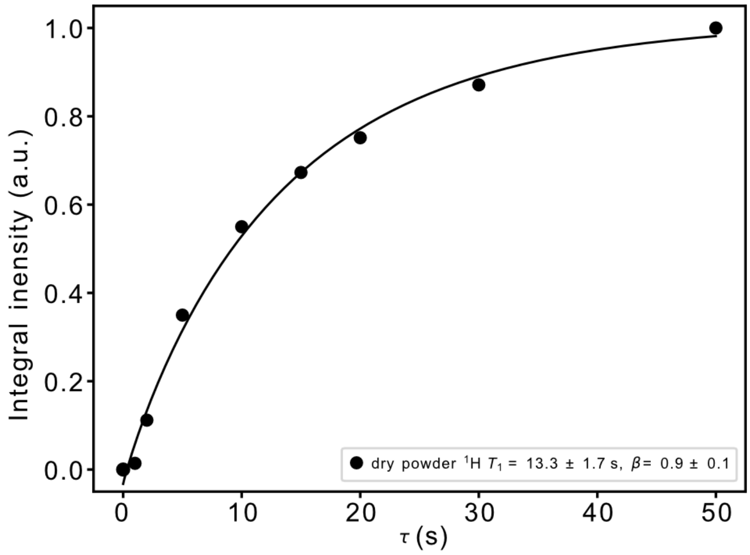

FIGURE S2. ^{1}H detected ^{1}H saturation recovery data of dry EHEC2 powder at T = 100 K, MAS = 8 kHz.

Supplementary discussion, swelling in D_2O/H_2O radical solutions

From SEM pictures such as Fig. S1 we know that intact EHEC particles are big, roughly 20-100 µm and typically shaped as cylinders with tendencies to aggregate. In this size range spin diffusion becomes irrelevant, since proton spin diffusion constants (D) are expected to be around 0.8×10^{-15} m^2/s in proton rich solids under conventional MAS solid-state NMR conditions[1]. The distance (r) that polarization diffuses during the time (t) is:

$$r = \sqrt{Dt}$$

And since practically all our relaxation delays are <12 s, polarization never diffuses longer than ~100 nm. So, in systems of this size spin diffusion becomes irrelevant. Instead, we claim that it is the swelling properties of EHEC in the radical solution that determines what DNP-enhancements, and longitudinal build-up times we observe. To theoretically investigate this further, we use a simple biexponential model to generate synthetic relaxation data, modelling rapidly relaxing (swelled) EHEC and non-swelled EHEC that relaxes with the T_1 of the dry powder (Fig. S2).

$$I(t) = I_{0sw}\left[f_{sw}\left(1 - e^{-t/T_{1sw}}\right)\right] + I_{0dry}\left[(1 - f_{sw})\left(1 - e^{-t/T_{1dry}}\right)\right]$$

We assume that EHEC which is swelled in the radical solution will have the same buildup time (T_{1sw}) as the radical solution part and also eventually reach the same equilibrium polarization (I_{0sw}), several ten times the normal Boltzmann distribution. Instead, the non-swelled part will buildup with the normal T_1 relaxation time of the dry EHEC powder, here referred to as (T_{1dry}) and eventually reach one Boltzmann distribution. Below we generate synthetic relaxation data sets for a cylindrical system where the outer layers become increasingly swollen in the radical solution. We calculate the fraction of swollen EHEC (f_{sw}) and generate the synthetic data sets. We then fit the synthetic data set with the stretched exponential function as we do for our real experimental data sets. This way we can learn what to expect from our experimental data sets for various degree of swelling.

In the next figure (Fig. S3) we show the results of fitting the synthetic data sets. EHEC is modelled as a small cylinder with 25 µm radius, swelling of the EHEC in the radical solution proceeds from the surface of the cylinder and inwards T_{1sw} is set to 3.6 s for the microwaves on scenario and to 4.2 s for microwaves off scenario, this based on the experimentally observed buildup times on the formate carbonyl signal (Fig. 2b & c in the main text). I_{0sw} is set to 60 in the microwave on case and to 0.5 when the microwaves are turned off. These values are based on the experimentally observed DNP-enhancement calculated as the ratio of peak integrals from the spectra acquired with microwaves turned on and off. This enhancement was around a factor of 120, and we a assume a depolarization[2] due to AMUPol usage down to 0.5 Boltzmann distributions in the microwave off case, so I_{0sw} in the microwave on case should be about 60.

Simulating swelling and relaxation in EHEC

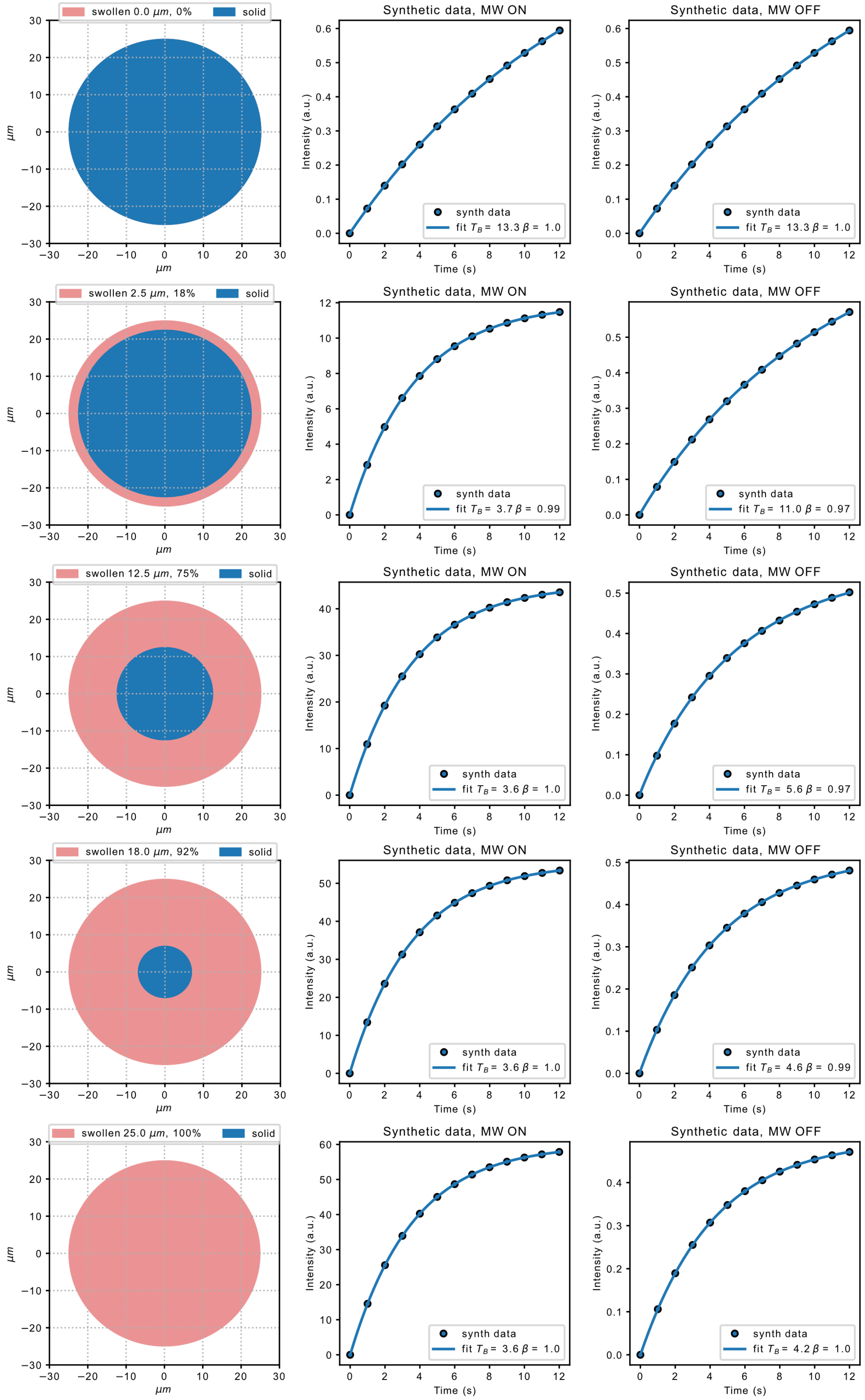

FIGURE S3. Fitting of synthetic relaxation datasets, generated with the biexponential model for swelling of the EHEC. The curves in the middle column illustrates that already at 2.5 μm/18% vol. of swelling, the relaxation becomes dominated by the enhanced, swelled part and the fitted T_B (T_{DNP}) value approaches that of the radical solution. The rightmost column shows that when the microwaves are turned off, complete swelling of the EHEC is necessary for the buildup time observed on the EHEC to equal that of the radical solution. This proves that for the relaxation curves of CO and C1 to be equal in Fig. 2c in the main text, EHEC needs to be fully swelled.

Properties of the z-filter and selective pulses

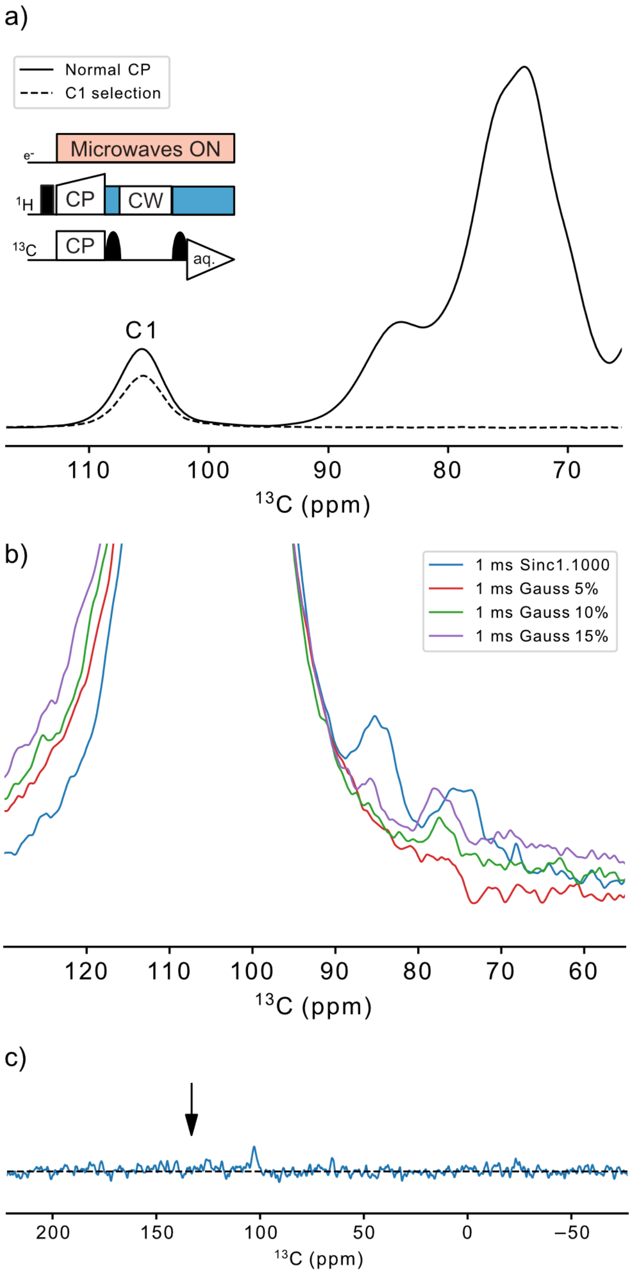

FIGURE S4. Properties of the z-filter and selective pulses. a) Comparison of the DNP-enhanced ^{13}C CP/MAS spectrum and the CP + z-filter part (pulse sequence shown) of the transfer experiment. The dashed spectrum shows C1 selection, the intensity of the C1 signal is ~50% of that in the CP/MAS spectrum. This is most likely due to relaxation during the two selective pulses in the z-filter. b) Artefact excitation from different soft pulses tested in the z-filter. c) Control experiment in which the selective pulses in the z-filter have been positioned +30 ppm relative to the C1 signal. As can be seen some C1 artefact signal is generated. The purpose of this control experiment is to exclude that dipolar mediated transfer phenomena plays a role in the artefact signals, this since the z-filter basically is a DARR[3] type mixing element.

Tests of 1D selective sequences on 13C/15N isoleucine

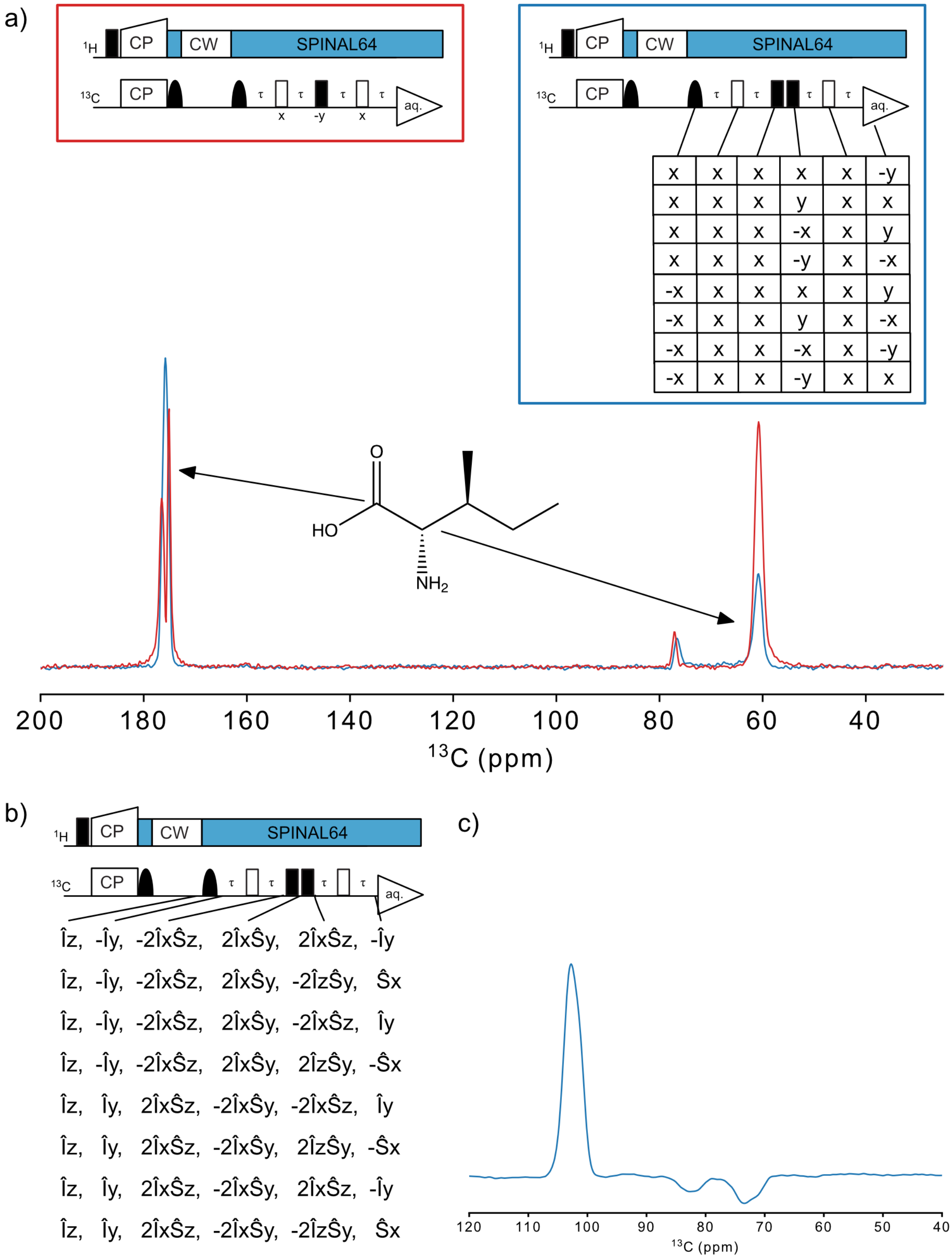

FIGURE S5. Tests of selective 1D sequences on isoleucine and EHEC. a) Room temperature transfer experiment tests of ^{13}C/^{15}N enriched isoleucine. Here it is easy to select the well-resolved CO signal at 176 ppm and transfer to C5 with perfect echo (red) double-quantum selective phase cycle (blue) experiment. b) Product operators at different positions in the pulse program, notice only transfer to S-spin/spin 2 for half of the scans, in good agreement with peak intensity in a) panel. c) Test of blue pulse sequence on EHEC2, here under DNP-conditions, the phase cycle fails to suppress artefacts and generate a lot of artefact signal, hence the simpler perfect-echo was chosen for transfer.

C2 quantification spectra

EHEC 1, 2048 scans

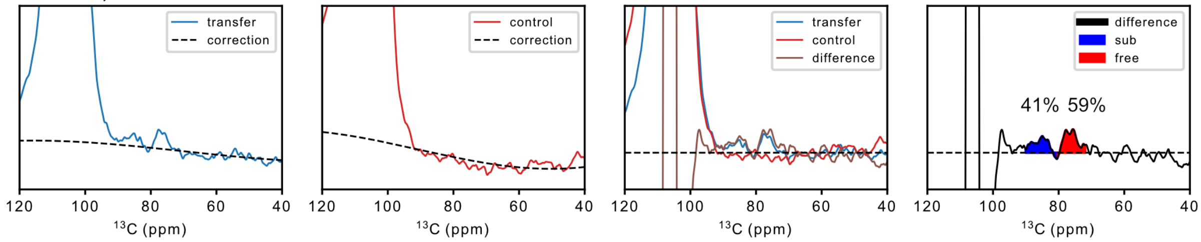

EHEC 1, 2048 scans

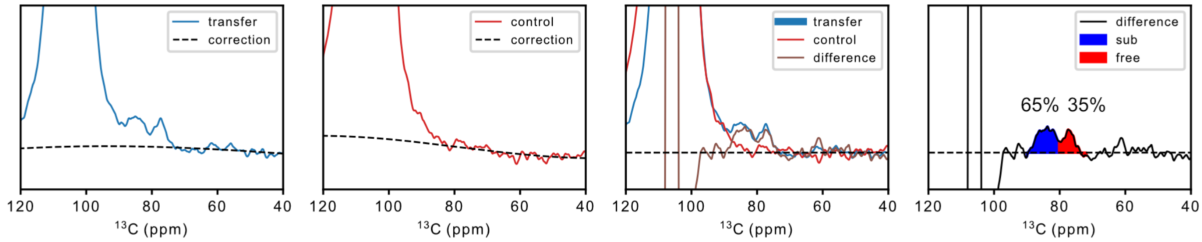

EHEC 1, 4096 scans

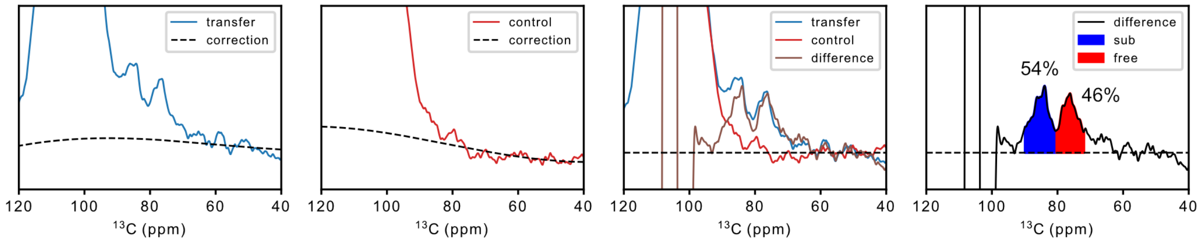

EHEC 2, 2048 scans

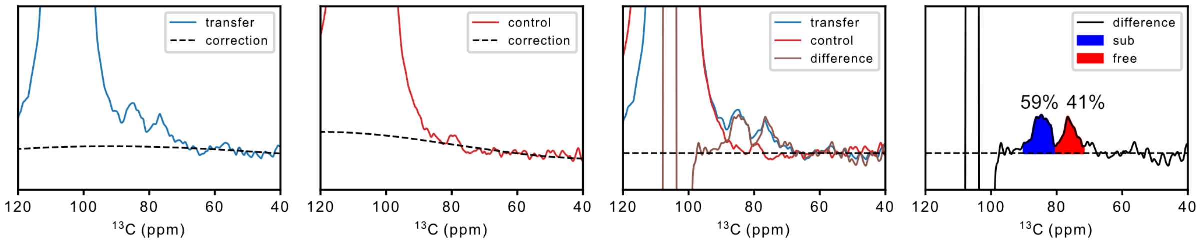

EHEC 3, 2048 scans

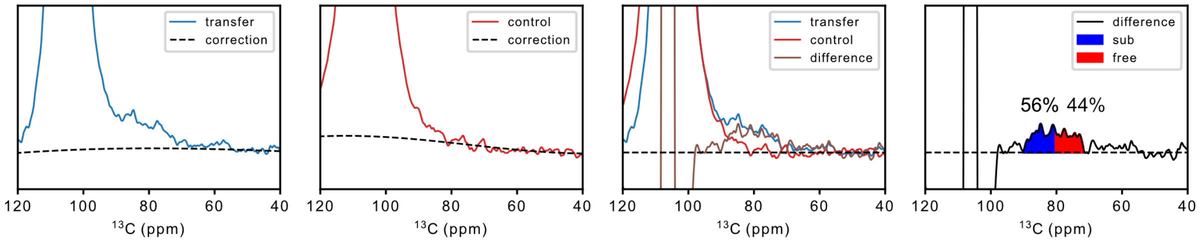

EHEC 3, 4096 scans

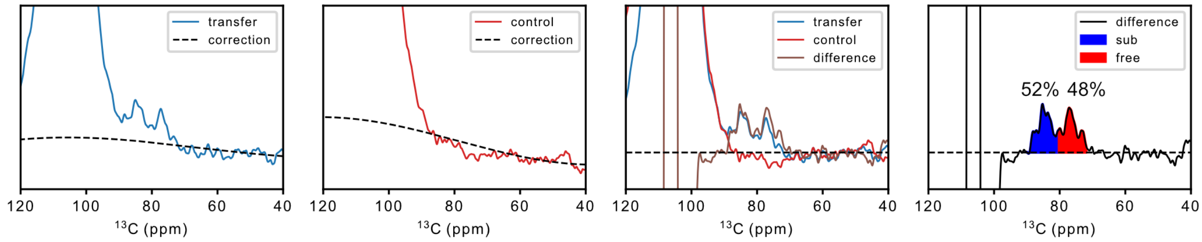

FIGURE S6. DNP-enhanced ^{13}C correlation spectra from the 1D, selective transfer experiments used for C2 quantification, tested on the different EHEC samples. Column one, shows the spectra from the actual transfer experiment with the baseline correction used. Second column shows the spectra from control experiments where hints of artefact excitation at 80 ppm can be seen, baseline correction is shown also here. The third column shows the subtraction/difference spectrum. The fourth column shows the integrated areas for C2 quantification and corresponding C2 substitution degrees in percent, the values that underlies Table 1 in the main article.

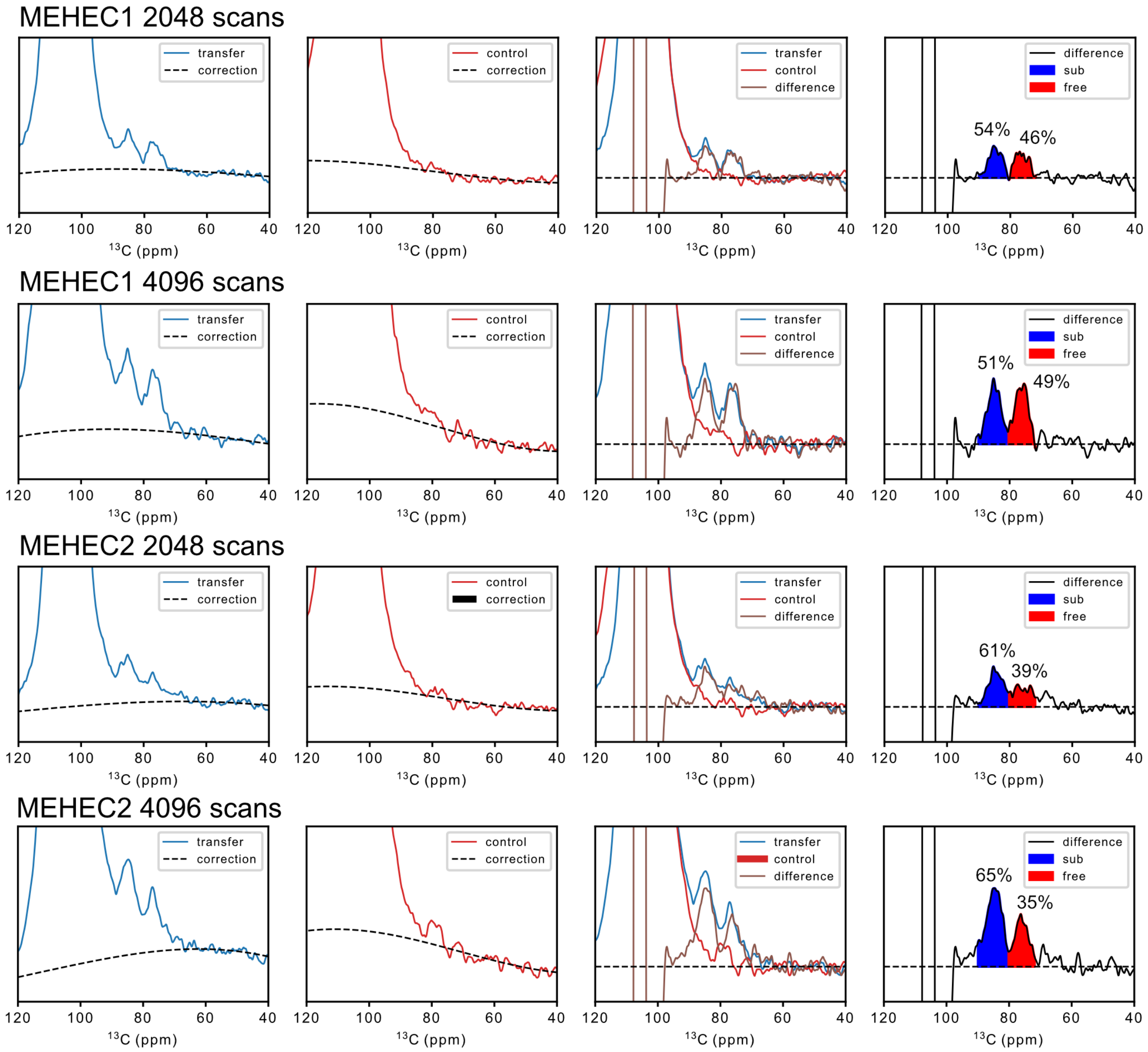

FIGURE S7. Spectra from the 1D correlation experiment, same as Figure S6 but here for the MEHEC samples.

Further example, baseline correction of 1D correlation spectrum

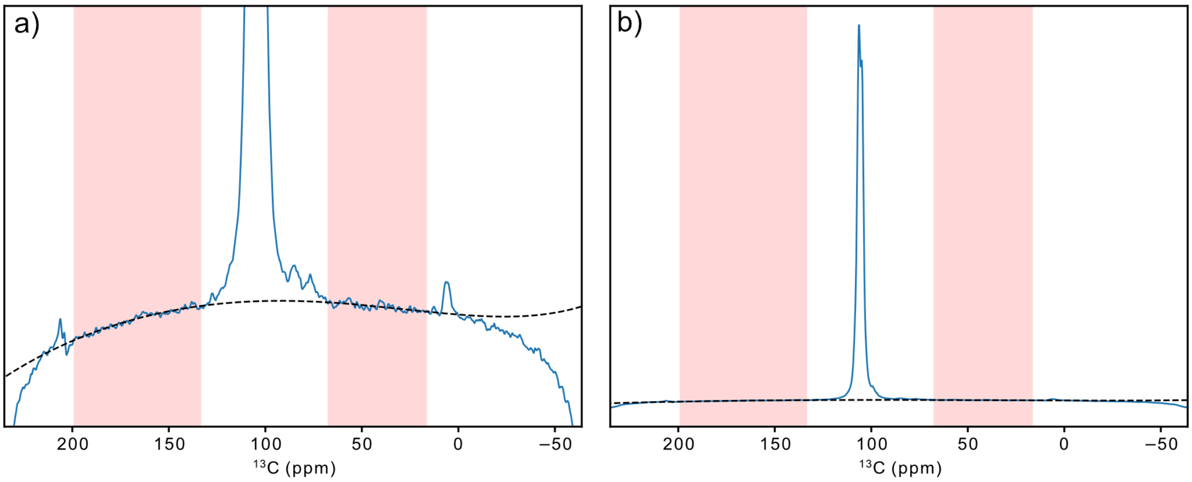

FIGURE S8. Example of baseline correction of the spectrum from the 1D transfer experiment of the EHEC2 sample. a) Close up view, fifth order polynomial (dashed line) is fitted to the red marked regions between the large C1 signal and the first spinning sidebands and is used as baseline correction. b) Full view of the spectrum.

References

1. Clauss, J., Schmidt-Rohr, K. Determination of domain sizes in heterogeneous polymers by solid-state NMR. *Acta Polymer* **44**, 1-17 (1993).

2. Mentink-Vigier, F. *et al.* Nuclear depolarization and absolute sensitivity in magic-angle spinning cross effect dynamic nuclear polarization. *Phys. Chem. Chem. Phys.* **17**, 21824–21836 (2015).

3. Takegoshi, K., Nakamura, S. & Terao, T. 13C±1H dipolar-assisted rotational resonance in magic-angle spinning NMR. *Chemical Physics Letters* 7 (2001).
